# Capillary Hysteresis in Neutrally Wettable Fibrous Media: A Pore Network Study of a Fuel Cell Electrode

**DOI:** 10.1007/s11242-017-0973-2

**Published:** 2017-12-01

**Authors:** T. G. Tranter, J. T. Gostick, A. D. Burns, W. F. Gale

**Affiliations:** 1 Department of Chemical Engineering, University of Waterloo, 200 University Ave. W, Waterloo, ON USA; 2 Centre for Integrated Energy Research, University of Leeds, Leeds, LS2 9JT UK

**Keywords:** Hysteresis, Capillary pressure, Fuel cell, Relative diffusivity

## Abstract

Hysteresis in the saturation versus capillary pressure curves of neutrally wettable fibrous media was simulated with a random pore network model using a Voronoi diagram approach. The network was calibrated to fit experimental air-water capillary pressure data collected for carbon fibre paper commonly used as a gas diffusion layer in fuel cells. These materials exhibit unusually strong capillary hysteresis, to the extent that water injection and withdrawal occur at positive and negative capillary pressures, respectively. Without the need to invoke contact angle hysteresis, this capillary behaviour is re-produced when using a pore-scale model based on the curvature of a meniscus passing through the centre of a toroid. The classic Washburn relation was shown to produce erroneous results, and its use is not recommended when modelling fibrous media. The important effect of saturation distribution on the effective diffusivity of the medium was also investigated for both water injection and withdrawal cases. The findings have bearing on the understanding of both capillarity in fibrous media and fuel cell design.

## Introduction

The capillary behaviour of multiphase systems in porous materials is of interest to many disciplines ranging from oil recovery in reservoirs Patzek (2001) to aiding the design and structure of pharmaceutical products Gladden et al. (2004). It is also of particular importance to the engineering of fuel cells, as phase interaction and distribution influences the performance of the cell by limiting mass transport Jiao and Li (2011). Multiphase modelling is a key focus of fuel cell research but relies on relationships between the capillary pressure, $P_\mathrm{C}=P_\mathrm{nwp}-P_\mathrm{wp}$, where $\mathrm{nwp}$ and $\mathrm{wp}$ refer to the non-wetting and wetting phase, and saturation, *S*, referring to the fraction of pore space occupied by water. The $P_\mathrm{C}$-*S* relations have been experimentally gathered for the fibrous gas diffusion layer (GDL) component of the electrode and display a somewhat puzzling hysteresis Gostick et al. (2009), whereby positive capillary pressure is required to inject water and negative capillary pressure is required to withdraw it. The processes both occur in an operating fuel cell: injection of water occurs from the reaction sites that lie at one side of the GDL and withdrawal is equivalent to drying which occurs in regions of the cell subject to high gas flow rate at the opposing side. This study aims to reproduce the $P_\mathrm{C}$-*S* relations for both water injection and withdrawal and investigate their impact on saturation distribution and consequently diffusive mass transport.

In the capillary-fingering flow regime, commonly occurring in fuel cells Sinha and Wang (2007), fluids displace one-another by a series of capillary-controlled menisci movements, widely termed drainage and imbibition. Drainage refers to the displacement of a fluid that preferentially wets the solid structures of the pore space by a non-wetting fluid and imbibition is the reverse. These terms are often misused and in the present work are actually ambiguous as neither fluid displays preferential wetting characteristics. However, for convenience, we follow convention and specify water as the non-wetting phase for the definition of capillary pressure.

It is common to view a porous media as a bundle or network of cylindrical tubes Dullien (1991). This conceptual picture leads directly to the following analytical relationship between pore size, *r*, and capillary pressure, $P_\mathrm{C}$ Washburn (1921), widely known as the Washburn equation or the Lucas–Washburn equation:

1 $$\begin{aligned} P_\mathrm{C} = -\frac{2\sigma \hbox {cos}(\theta )}{r} \end{aligned}$$

where $\sigma$ is the surface tension of the fluid–fluid interface, and $\theta$ is the contact angle between the two-phase contact line and the solid surface, typically measured through the non-wetting phase. Equation 1 is valid for straight, circular capillary tubes of fixed radius. This relation has led to the unfortunate convention that a contact angle of 90$^\circ$ indicates a wholesale switch in wettability, which stems from the fact that the $\hbox {cos}(\theta )$ term changes sign at 90$^\circ$, changing the capillary pressure from positive (drainage) to negative (imbibition). However, many materials can be classified as neutrally or intermediately wettable and this poses a problem for the above definitions of drainage and imbibition Anderson (1986), Anderson (1987). The traditional concepts of drainage and imbibition can only be expected in strongly wetting systems, where the contact angle through the wetting fluid is less than about 60$^\circ$ Shirtcliffe et al. (2006) and Sahimi (2011).

Fuel cell GDLs are highly porous sheets made from randomly laid carbon fibres with approximately 10 $\upmu \hbox {m}$ diameters Park et al. (2012). The main role of the GDL is to facilitate oxygen diffusion from the gas supply channels to the catalyst sites in the cell. To prevent flooding, GDLs are typically coated with a polymer such as poly-tetra-fluoro-ethylene (PTFE) to enhance their hydrophobicity, and limit the spreading of liquid water throughout the pore space. Because the base material is carbon, which can have a wide range of contact angles for water air systems Tadros et al. (1979), Easton and Machin (2000) and Parry et al. (2010) but is generally accepted to be lower than 90$^\circ$, untreated GDLs are widely considered to be hydrophilic by the convention of Eq. 1. By the same logic, the addition of PTFE, with a contact angle of 108$^\circ$ Owens and Wendt (1969), is then supposed to render them at least partially hydrophobic. Some early experimental evidence supported the picture of GDLs having a partially hydrophilic component Gostick et al. (2008), since it was found that removal of water from the GDL required application of negative capillary pressures. Subsequent measurements Fairweather et al. (2007), Harkness et al. (2009) and Gostick et al. (2009) showed conclusively that positive pressures were required to inject liquid water into GDLs, and negative pressures were required to withdraw it.

Together these measurements demonstrated an extreme hysteresis, where neither water nor air will spontaneously imbibe, that was difficult to rationalise. Weber (2010) and Weber et al. (2014) explain the data using contact angle hysteresis, which is the only mechanism by which Eq. 1 changes sign, meaning that the water becomes the wetting fluid when trying to withdraw it. Cheung et al. determined the pore-size distribution from mercury intrusion data, then advancing and receding contact angles were used as adjustable parameters to fit the water injection and withdrawal behaviour within the context of Eq. 1Cheung et al. (2009). An advancing contact angle of 92$^\circ$ was required, while the receding angle was just 52$^\circ$. Not only is a 40$^\circ$ swing between advancing and receding values unusually wide, but the fitted withdrawal contact angle is far below the expected values for materials present in the GDL. These studies highlight major limitations of the simple cylindrical tube model. In addition, Gostick’s pore network model with realistic pore-size distribution showed that a contact angle of around 130$^\circ$ would be required to fit water intrusion data when using Eq. 1 Gostick (2013). These results signalled an additional problem with the existing understanding of capillarity in GDLs, namely that GDLs appear much more hydrophobic/hydrophilic upon water injection/withdrawal than expected.

Harkness et al. first suggested the possibility that the behaviour of water in GDLs could be better described by accounting for the converging–diverging geometry of throats formed by fibres Harkness et al. (2009), in contrast with the assumption of parallel walls implicit in Eq. 1. The capillary behaviour for converging–diverging throats can be adequately modelled as a meniscus moving through a three-dimensional toroidal ring. The model was first proposed by Purcell to account for hysteresis in capillary pressure for drainage and imbibition in rock formations Purcell (1950) and was extended from a formula derived by Crisp and Thorpe (1948) for parallel cylinders when modelling the water repellent properties of insect hairs. The model is henceforth referred to as the toroidal model and is directly applicable to fibrous media with constant fibre diameter and constant wetting properties. The model was explored by Mason and Morrow (1994) who provide the formula for the critical entry pressure:

2 $$\begin{aligned} P_\mathrm{C}=\frac{-2\sigma }{r} \cdot \frac{\hbox {cos}(\theta -\alpha )}{1+\frac{R}{r}(1-\hbox {cos}(\alpha ))} \end{aligned}$$

where *r* is half the minimum fibre spacing or pore/throat radius, *R* is the fibre radius and $\alpha$ is the filling angle, defined as zero when the interface reaches the smallest constriction or the apex. The filling angle modifies the capillary pressure and has minimum and maximum corresponding to threshold pressures for injection and withdrawal of the interface. At the apex, Eq. 2 reduces to Eq. 1, but importantly, this is not the point at which maximum interface curvature occurs, as shown in 1.

**Fig. 1:**
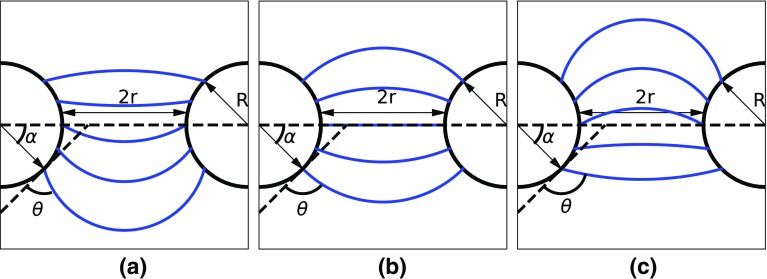
Shape of the meniscus as invading phase moves upwards through the torus with key model parameters. Contact angles through invading phase, $\theta$ $=$ **a** 60$^\circ$, **b** 90$^\circ$ and **c** 120$^\circ$. All scenarios clearly show an inflection of the meniscus curvature signifying a switch in the sign of the capillary pressure from negative to positive. This inflection is predicted to occur for all contact angles by the model with varying filling angle, $\alpha$. Here, a two-dimensional cross-section is shown for simplicity, but Eq. 2 is valid for three-dimensional rings

The toroidal model predicts that the meniscus will advance past the throat apex before reaching the critical or breakthrough pressure, increasing curvature due to the locally diverging geometry, which could explain the apparent extra hydrophobicity/hydrophilicity of GDLs. The model was partially validated using pore network modelling Gostick (2013), which was able to match both mercury and water injection using the same pore and throat size distribution and typical contact angles of 140$^\circ$ and 108$^\circ$, respectively. More importantly, and the focus of the present study, the toroidal model also applies to the receding meniscus to achieve breakthrough of air in neutrally wetting fibrous media. This results in negative capillary pressures for intermediate contact angles and, we posit, explains the extreme capillary pressure hysteresis observed in GDLs.

The present work demonstrates that the toroidal model for capillary pressure can simultaneously explain both the high hydrophobicity of water injection and the apparent hydrophilic behaviour of water withdrawal from GDLs using a simple pore network modelling approach. This is achieved by also considering the interactions of the meniscus with additional solid features and fluid topology. The effect of differing saturation distributions on the relative transport through the network is also investigated and shown to be important. All network generation and simulations were performed using OpenPNM (Gostick et al. 2016). Although the application used to demonstrate the model is based on one specific material used in fuel cells, the conclusions drawn are not particular to fuel cells and should apply to fibrous media in general providing the distribution of fibre radii is sufficiently narrow.

## Methodology

Throughout the rest of this paper, the terms drainage and imbibition are avoided entirely, since they have very specific connotations, as outlined in the Introduction. Instead, the terms injection and withdrawal are used, referring to the liquid water phase. This convention reinforces the fact that truly wetting and non-wetting behaviour by either phase is not observed in the present system.

### Capillary Pressure Model

**Fig. 2:**
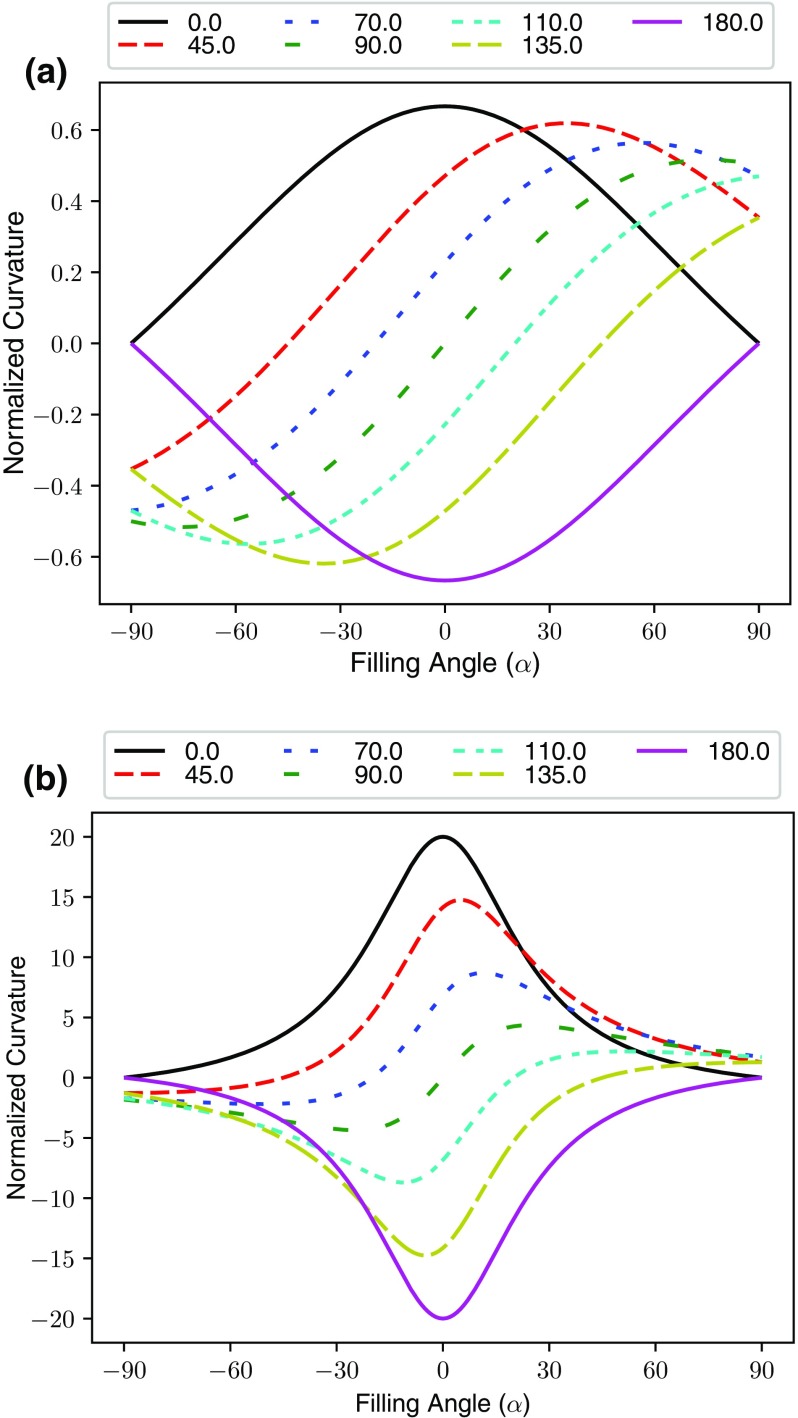
Dependence of normalised meniscus curvature on the filling angle for **a** $r/R = 3$, a typical value for the present study and **b** $r/R = 0.1$, for comparison with Mason and Morrow (1994), $\theta$ is measured through the defending phase. A striking difference between the higher and lower throat to fibre radius ratio is the magnitude of the maximum curvature which is almost independent of contact angle for higher ratios

In network modelling, the maximum capillary pressure is used as the throat entry pressure for the invading phase during percolation simulations, whereby fluid breaks through a throat constriction and enters the neighbouring pore. The toroidal model for capillary pressure, Eq. 2, accounts for the converging–diverging nature of fibrous material structures by imagining the meniscus passing through the centre of a torus. Figure 1 shows that an inflection of the interface from concave to convex is observed, as the meniscus moves through the constriction. This signifies that positive pressure is required in the invading phase to transition through the constriction. The filling angle ($\alpha$) at which the maximum and minimum meniscus curvatures occur was shown by Mason and Morrow (1994) to be:

3 $$\begin{aligned} \alpha ^\mathrm{max}=\theta - \hbox {sin}^{-1} [\hbox {sin}(\theta )/(1+r/R)] \end{aligned}$$

4 $$\begin{aligned} \alpha ^{min}=\theta -180 + \hbox {sin}^{-1} [\hbox {sin}(\theta )/(1+r/R)] \end{aligned}$$

where $\theta$ is measured through the defending phase. Mason and Morrow showed that curvature depends on the filling angle and that solutions are symmetrical contact angles $\theta$ and $180 - \theta$ about $\alpha$ and zero curvature. In other words: Eq. 3 $+$ 4 is constant, when no contact angle hysteresis occurs. Figure 2a shows the normalised curvature as a function of filling angle for a typical GDL throat radius to fibre radius ratio, r/R, of 3. The curves shown are quite different to those presented by Mason and Morrow who use a ratio of 1/10 which has been re-produced for convenience in Fig. 2b. The main difference is that the magnitude of the maximum and minimum normalised curvatures show little dependence on contact angle in Fig. 2a when r/R is large. Therefore, for throats that are of a comparable size to the fibres and larger, the effect of contact angle on the characteristic entry pressure is almost negligible. According to the geometrical properties of the GDL, the addition of PTFE to increase liquid contact angle should have no effect on the injection and withdrawal pressures of water; in direct conflict with experimental observations that showed the entire injection and withdrawal loops shift to higher pressures in PTFE-treated GDLs Gostick et al. (2009).

**Fig. 3:**
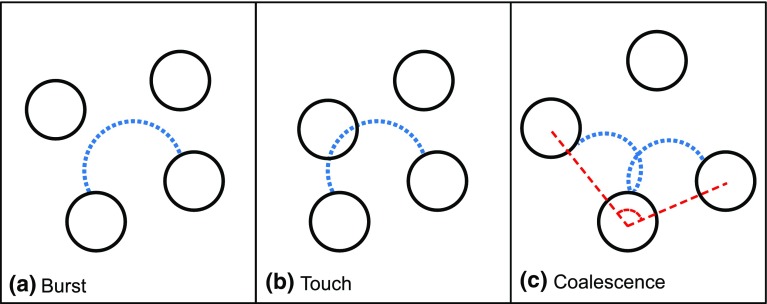
Three pore invasion mechanisms are implemented using the toroidal model: **a** Burst occurs when the meniscus reaches maximum curvature corresponding to a filling angle of $\alpha ^\mathrm{max}$. **b** A solid feature is touched by the meniscus before maximum curvature and pore penetration occur, the inscribed sphere radius is compared to the penetration depth to determine whether this condition is met. **c** Coalescence of menisci in throats that share a pore occurs when there is an intersection of their spherical caps inside the pore. Coalescence results in cooperative pore filling as described in Sect. 2.4.3

To address this limitation of the model, we note that although the maximum capillary pressure is essentially unchanged with contact angle, the maximum and minimum filling angle where breakthrough occurs does change. This has bearing on the actual peak entry pressure when one considers that menisci are not free to advance to their maximal filling angle, but may actually touch surrounding pore walls before reaching the critical pressure. Moreover, simultaneously growing menisci in neighbouring throats could also coalesce inside a pore if they touch before reaching the critical curvature or another solid. These invasion mechanisms have been modelled in two dimensions with arrays of circular discs Cieplak and Robbins (1990) and Chapuis et al. (2008) and are termed “burst” for standard entry where capillary pressure reaches the maximum stable interface pressure, “touch” for the touching of additional solid features and “coalescence” for fluid–fluid interaction. The three invasion mechanisms are illustrated in two dimensions in Fig. 3.

**Fig. 4:**
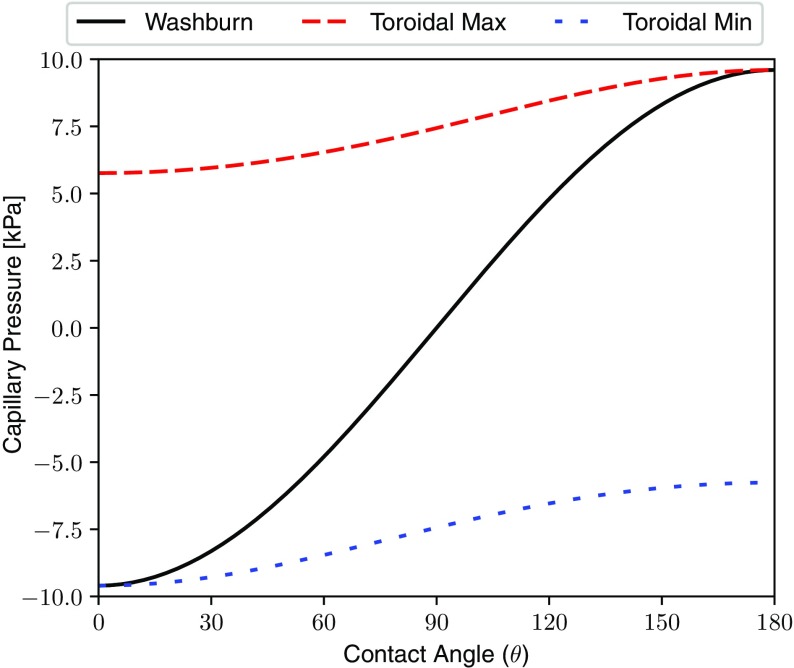
Comparison of the Washburn and toroidal models for capillary pressure over the full range of contact angles with $r/R = 3$. The toroidal model predicts that maximum pressure in a throat is always positive, irrespective of contact angle

Touching of solid features by the growing menisci is accounted for when using the toroidal model by comparing the pore-penetration depth of the menisci with the diameter of an inscribed sphere within the pore being invaded. If the maximum distance from the throat centre along the meniscus exceeds the sphere’s diameter, at a filling angle lesser than the critical angle required for “burst” entry, the pressure at this point is used instead. Coalescence of simultaneously growing menisci within a pore is also accounted for by considering the intersection of all growing menisci simultaneously penetrating into the pore. The coalescence process can also be described as cooperative pore filling and the method for calculating the capillary pressure at which a coalescence event occurs is described in Sect. 2.4.3.

Accounting for the “touch” and “coalescence” conditions, and implementing them into the percolation algorithm is non-trivial as the throat entry pressure is now, not only dependent on the throat size, but also the pore size and neighbouring throat occupancy. By definition, a throat cannot have a radius larger than that of the pores it connects with. However, if the pore has a high aspect ratio, the inscribed sphere diameter used for the “touch” condition can be smaller than some of the larger connecting throat diameters. In anisotropic media like fibrous mats such as GDLs, this pore filling mechanism becomes increasingly important.

Incorporation of filling angle and meniscus advancement into the calculation of invasion capillary pressure provides an explanation of the asymmetry between the average injection and withdrawal pressures when contact angle is not 90$^\circ$. For illustration purposes, consider a meniscus that only reaches a filling angle of 45$^\circ$ in all throats before touching a solid feature or additional fluid meniscus within the connecting pore. Dropping a vertical line at 45$^\circ$ into Fig. 2a intersects the normalised curvatures at very different values compared with the maximum curvatures, thereby giving rise to the shift in capillary curves seen with the addition of PTFE (i.e. altered contact angle). Thinking of the situation the other way, for a given curvature or pressure difference, a wetting phase will penetrate further into a pore, compared with a non-wetting phase, thereby increasing the likelihood of touching a pore wall or other menisci, resulting in lesser invasion pressure differences.

Figure 4 illustrates the difference between the two pore-scale capillary pressure models described by Eqs. 1 and 2 over the full range of contact angles. It is clearly shown that the toroidal model predicts that the maximum meniscus curvature is positive regardless of the contact angle, i.e. positive pressure is always required in the invading phase for invasion to occur. Therefore, for wetting phases, where the contact angle measured on a flat surface is less than 90$^\circ$, the error introduced by using the Washburn equation for fibrous geometry is large. However, the toroidal model is expected to breakdown for the withdrawal of highly non-wetting fluids or injection of highly wetting fluids, where corner film flow is expected to occur. For injection of perfectly non-wetting fluids, the toroidal and Washburn equations predict similar capillary pressures. This is a critical point since it means that in highly non-wetting systems, such as mercury intrusion or water in glass, the impact of the toroidal throat shape is negligible and the Washburn approximation is valid.

The aim of the present study is to determine whether or not the toroidal model can predict the observed extreme hysteresis in the capillary behaviour of neutrally wettable fibrous media using sensible contact angles, while also matching experimentally measured gas phase diffusivity in partially water filled samples. Three numerical cases are investigated matching experimental capillary pressure data for an uncompressed GDL material, the details of which are summarised in Table 1. The toroidal model is used as the pore-scale capillary pressure model without applying contact angle hysteresis for Case A. For contrast, the Washburn model is used applying moderate and extreme contact angle hysteresis in Case B and C in order to match the experimental data.

**Table 1:** Details of the capillary pressure models for each numerical case

Case	$P_\mathrm{C}$ model	Water/air $\theta$	Hysteresis $\theta$
A	Eq. 2	110/70	0
B	Eq. 1	110/110	40
C	Eq. 1	150/130	100

### Network Generation

It was previously demonstrated that realistic fibre geometries could be generated using Voronoi diagrams and Delaunay tessellations to produce spatially correlated random networks. These networks were verified by the fit to water injection and mercury intrusion data Gostick (2013), and have also been used to study the effects of compression on multiphase flow in GDLs Tranter et al. (2016). A considerable advantage of using a Voronoi diagram to create the network is that correlation between pore size and location arises naturally. The technique also allows for reproduction of key features of the materials being studied, such as high porosity and connectivity. Anisotropy is also easily reproduced by scaling the pore coordinates and vertices defining the fibre locations in a particular direction.

**Table 2:** Probability functions applied to the networks to adjust pore densities to negate and allow for porosity gradients in the in-plane (IP) and through-plane (TP) directions, respectively

Direction	Probability function	*a*	*b*
IP	$p = (m^a +(1-m)^a + b)/(1 + b)$	35	0.2
TP	$$p = 1-(m^a + (1-m)^a + b)/(1 + b) $$	10	0.5

For the present study, a domain of size 750 $\times$ 750 $\times$ 500 $\upmu \hbox {m}$ was initially populated with 2500 pores according to the pore placement probability functions presented in Table 2 for each principle direction where p is the probability that a pore is placed at relative position m in the domain and a and b are parameters that adjust the probability to apply porosity gradients across the domain. As GDLs are paper-like and characterised by being thin and planar, the terms in-plane (IP) and through-plane (TP) are used to describe transport along the sheet surface direction and normal to it. The intended effect of the IP probability function is to negate the effect of the Voronoi diagram generation which results in larger pores at the domain edges Gostick (2013). The TP probability function decreases the pore-density at the top and bottom surfaces of the domain, creating larger pores. This is intended to simulate the observed through-plane porosity gradients commonly found in GDLs Fishman and Bazylak (2011), where regions near the top and bottom surfaces are more porous.

**Fig. 5:**
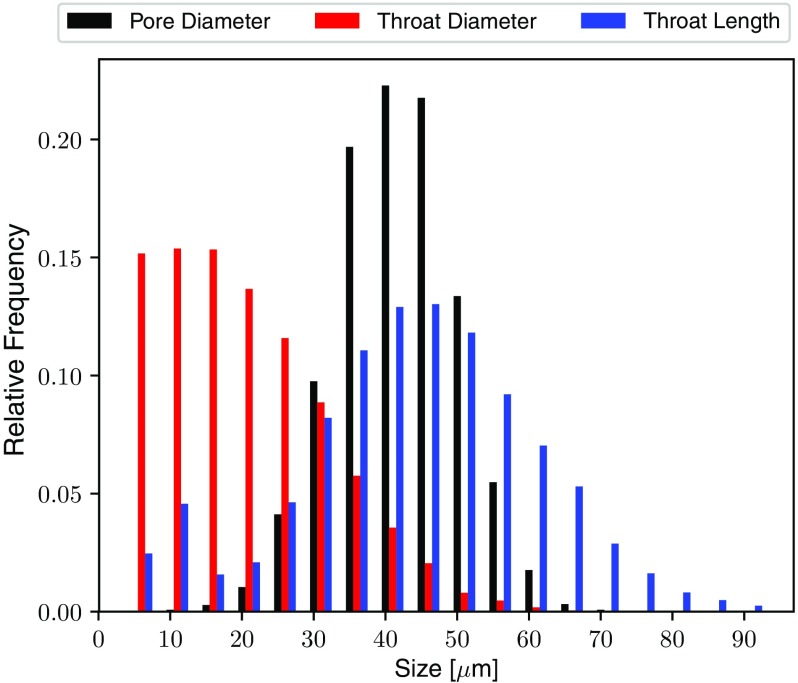
Various size distributions for the networks generated from the Voronoi Diagram representing fibres and Delaunay Tessellation giving connections

**Fig. 6:**
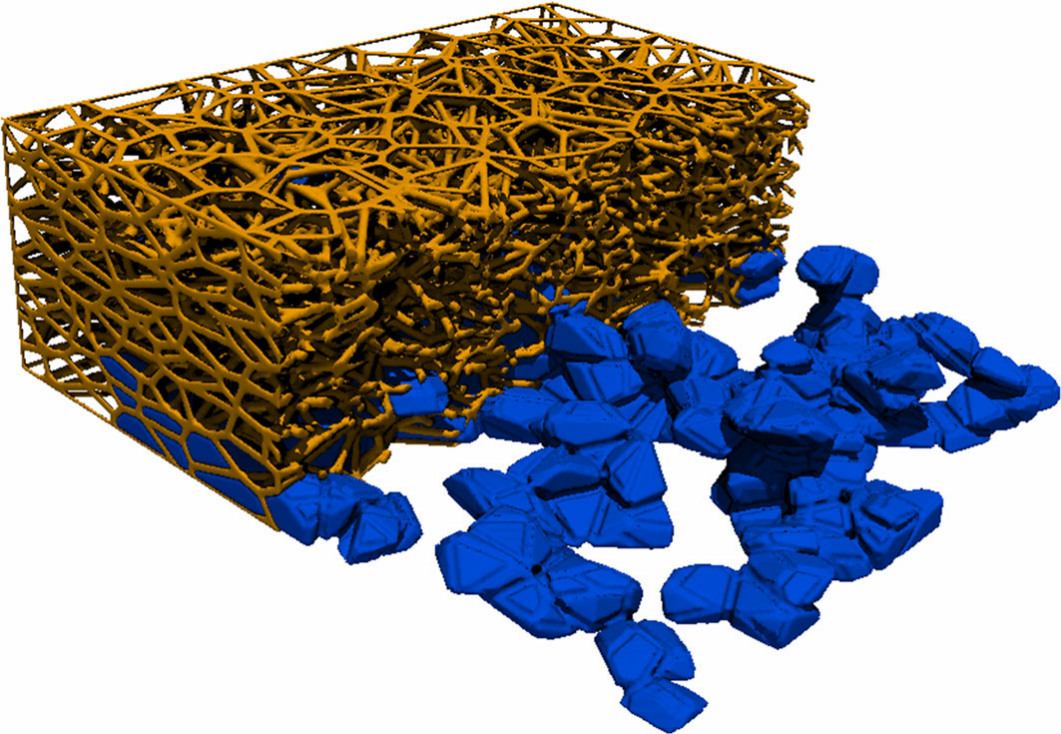
Visualisation of the anisotropic fibrous geometry created from the Voronoi diagram. Anisotropy is introduced by scaling the vertices at each end of the fibre in the through-plane direction. Porosity distributions are introduced by varying the pore placement density. The image has also been populated with water from the invasion algorithm and can be used for conducting other image based simulations

Connections between pores are defined using a Delaunay tessellation and the fibrous geometry is generated with the complimentary Voronoi diagram. To introduce anisotropy the pore coordinates and Voronoi vertices forming fibre intersections are scaled in the through-plane direction by a factor of 0.5, reducing the domain height to 250 $\upmu \hbox {m}$ and giving fibres an IP alignment. A 3D image is then created giving volume to the fibres and pore and throat sizes are determined utilising the special property of the Voronoi diagram, which is that regions between fibres are always convex hulls Gostick (2013) and Tranter et al. (2016). Important network sizes are shown in Fig. 5 and an image of the fibrous geometry which has been partially saturated is shown in Fig. 6. The pore sizes are generally larger than throat sizes by a factor of 2 or 3 which by traditional network standards is not large, and both are many times larger than the fibre size which is an important feature of the highly porous fibrous media, as discussed previously. Pore sizes in the present study compare well with sizes extracted from real GDL materials Luo et al. (2010) and Gostick (2017).

### Effective Transport Properties

PNMs were specifically conceived to solve two-phase transport problems with ease Fatt (1956). This is accomplished by determining the discrete configuration of the invading and defending phase using the appropriate percolation algorithm, then solving the system of linear equations for the transport property of interest for each phase separately like a resistor network.

#### Governing Equations

The diffusive conductance governing the transport of species between pores through the connecting throats is defined as:

5 $$\begin{aligned} g_D=\frac{cD_{ab} \phi _i}{l_i} \end{aligned}$$

where *c* is the molecular density of the gas, $D_{ab}$ is the binary diffusion coefficient of species a through stagnant b in open space and $\phi _i$ and $l_i$ are the throat cross-sectional area and length of the pore or throat respectively.

The diffusive conductance is then used to calculate the species concentration profile by solving a series of one-dimensional linear equations according to species conservation:

6 $$\begin{aligned} n_{a,ij}= g_d (x_{a,i}-x_{a,j}) \end{aligned}$$

where $n_{a,ij}$ is the molar flux between pores *i* and *j* and $x_a$ is the mole fraction of species *a*. The total diffusive conductance for a pore-throat-pore conduit is found by combining the individual conductance values like resistors in series, taking the pore radii and throat length as an appropriate length scale:

7 $$\begin{aligned} \frac{1}{g_{d,ij}} = \frac{1}{g_{d,i}} +\frac{1}{g_{d,t}} + \frac{1}{g_{d,j}} \end{aligned}$$

The effective diffusivity, $D_\mathrm{eff}$, of the network is found using Fick’s 1st Law:

8 $$\begin{aligned} N_a= \frac{cD_\mathrm{eff} A}{L} (ln(x_{a,\mathrm{in}})-ln(x_{a,\mathrm{out}})) \end{aligned}$$

where $N_a=\sum {n_i}$ for all pores at the boundary of the domain, *A* is the cross-sectional area of the domain normal to the flow, and L is the length of the domain between the boundaries.

The pore-throat-pore conduits with water in any of their elements are effectively excluded from the network by reducing their conductivity by 6 orders of magnitude. In this way, relative effective diffusivity can be calculated as follows:

9 $$\begin{aligned} D_r=\frac{D_{\mathrm{eff}(S)}}{D_{\mathrm{eff}(S=0)}} \end{aligned}$$

Similarly, the effective permeability of the medium can be found using a hydraulic conductance according to Hagen–Poiseuille flow:

10 $$\begin{aligned} g_h=\frac{\pi r_i^4}{32l_i \mu } \end{aligned}$$

where $r_i$ is the radius of the conduit and $\mu$ is the dynamic viscosity of the flowing fluid. The total hydraulic conductance for a pore-throat-pore conduit is found by combining the individual conductance values as per Eq. 7 and Darcy’s Law is used to calculate the permeability ($K_0$) of the medium:

11 $$\begin{aligned} Q=\frac{K_0 A(P_{in}-P_{out})}{L\mu } \end{aligned}$$

where *Q* is the volumetric flow rate through the medium, and $P_{in}$ and $P_{out}$ are the pressures at the inlet and outlet faces of the medium, respectively.

#### Network Sizing

Trial-and-error was used to tune the network by adjusting the number of pores in the domain, the degree of anisotropy, and the through-plane pore distribution. The simulations presented in Sect. 3.1 were compared with experimental data for Toray 090 with 20% PTFE treatment. Three matching criteria were necessary to obtain a realistic network, the water injection curve given by Gostick et al. (2009) which will be discussed later, the porosity of about 0.8 Rashapov et al. (2015b) and the absolute permeability which has been measured at about 1.5 $\times$ 10$^{-11}$ m$^2$ and 9 $\times$ 10$^{-12}$ m$^2$, for IP and TP directions, respectively Gostick et al. (2006).

Many pore-size distributions could result in similar capillary pressure curves, but additionally fitting permeability and porosity nearly assures geometrically representative size distributions Ioannidis and Chatzis (1993). Simulated network porosity is 0.83, permeability is 1.3 $\times$ 10$^{-11}$ m$^2$ and 7.8 $\times$ 10$^{-12}$ m$^2$ for IP and TP, respectively, which compares well with the literature. Absolute diffusivity is also calculated as 1.1 $\times$ 10$^{-5}$ m$^2$s$^{-1}$ and 5.2 $\times$ 10$^{-6}$ m$^2$s$^{-1}$ for IP and TP, respectively. These values also match well with the studies of TP diffusion Hwang and Weber (2012) and IP diffusion Tranter et al. (2017), Rashapov et al. (2015a) and Rashapov and Gostick (2016).

### Percolation Model

The toroidal model for capillary pressure applied to neutrally wetting fibrous media essentially predicts that the wetting phase acts like a non-wetting phase. Therefore, we hypothesise that both injection of water and withdrawal of water or injection of air should follow the same rules. Differences occur due to the formation of wetting films at the sub-pore-scale level, and these are accounted for by applying trapping to the water phase and snap-off to the air phase under water withdrawal.

#### Invasion Percolation

Both injection and withdrawal of water are simulated with a modified version OpenPNM’s invasion percolation algorithm Gostick et al. (2016). In addition, both water injection and withdrawal are simulated with bond percolation (controlled by throat sizes), where traditional imbibition algorithms use site percolation (controlled by pore sizes). The algorithm proceeds as follows:

1. Defending and invading phases are specified and the domain is initially filled with the defending phase.
2. Inlet pores are selected from the boundary face using every other pore and filled with the invading phase. These pores form the starting point for the invading cluster. Throats connected to the inlet pores are added to a dynamically updated queue that automatically sorts them based on entry capillary pressure. Either the bottom or top faces of the network are designated as boundary faces for water injection and withdrawal, respectively for results presented in Sect. 3.1 when matching capillary pressure data. For comparison, both top and bottom faces are designated as boundaries for both injection and withdrawal for results presented in Sect. 3.2 when simulating diffusivity. These conditions were all chosen to correspond with the experimental conditions.
3. At each invasion step, the throat with the lowest entry pressure (i.e. top of the queue) is invaded along with the connecting pore. All the newly accessible throats are added to the queue for the next step.
4. Clusters of invading phase may merge together and invasion proceeds until the domain is completely filled with the invading phase.
5. Trapping is then calculated as a post-process, as described in Sect. 2.4.2

Both injection and withdrawal of water are considered to be access limited. i.e. the fluid interface only invades pores connected to the invading cluster. However, snap-off essentially introduces additional inlets for invasion in the body of the domain. As will be discussed in the following sections, multiple types of invasion events can occur. This is handled automatically by the invasion algorithm which sorts potential invasion steps by capillary pressure. Therefore, multiple events may be added to the queue for the same set of pores and throats and the one with lowest absolute capillary pressure will be enacted with the rest ignored.

#### Trapping and Late Pore Filling

Trapping of the defending phase may occur when the invading phase completely encircles a pore or collection of connected pores currently occupied by the defending phase. For water injection, it is assumed that air maintains a continuous network via cracks and corners until the very end of the experiment where pressure increases and all air is squeezed from the network, as previous models have assumed Gostick (2013), Tranter et al. (2016). Therefore, the wetting phase (air) does not become trapped, in agreement with experimental observations García-Salaberri et al. (2015b) and García-Salaberri et al. (2015a), but water does upon withdrawal.

To model the squeezing of residual air within individual pores, a heuristic late pore filling model is employed that accounts for sub-pore-scale features filling after initial invasion of the pores:

12 $$\begin{aligned} S_\mathrm{res} = 0.25\left( \frac{P_\mathrm{C}^*}{P_\mathrm{C}}\right) ^{2.5} \end{aligned}$$

where $S_\mathrm{res}$ is the residual air fraction inside pores and $P_\mathrm{C}^*$ denotes the capillary pressure upon initial invasion. This heuristic model is usually introduced as a way to account for the gradual filling of pores at higher capillary pressures, but could equally be thought of as invading smaller pores in the traditional sense which do not form part of the main transport network.

In conjunction with ordinary percolation, trapping is typically applied as a post-processing step and identifies clusters of pores and throats that are both uninvaded and disconnected from the outlet face(s) of the domain. Previously, trapping models employed in conjunction with invasion percolation were performed after every step in the algorithm, making it quite difficult to develop highly efficient algorithms Sheppard et al. (1999). However, a fast invasion percolation algorithm with trapping was recently published by Masson (2016) and has been implemented in OpenPNM for the present study. The basic premise is to run the invasion percolation algorithm to completion without trapping and then run it backwards by reversing the invasion sequence and assessing the phase occupancy of the neighbours of each “uninvaded” pore. During percolation reversal, trapped clusters may grow and merge when unconnected with an outlet or sink. Once a path to an outlet is made, i.e. when a trapped cluster first meets a non-trapped cluster, the trapped cluster is fixed in size. This is the point in forward time invasion percolation that trapping first occurs and trapped clusters cannot reduce in size thereafter as phase change is not considered and water is assumed to be incompressible.

**Fig. 7:**
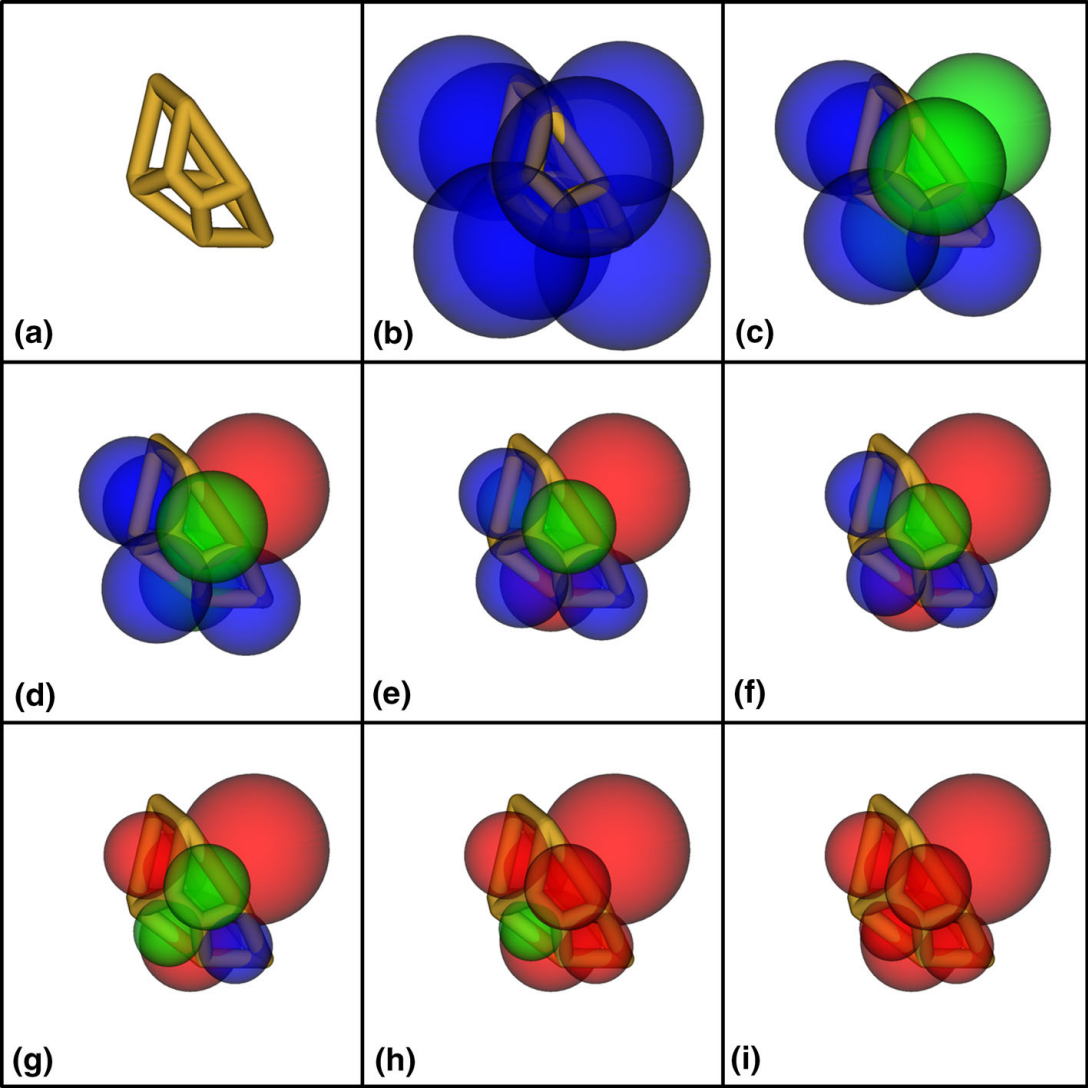
Cooperative pore filling sequence for a single pore in the network: **a** No phases present, **b** Pc $=$ 3000 Pa, invading phase present at every throat with no intersection (blue), **c** Pc $=$ 4000 Pa, menisci spheres begin to intersect (green) as filing angle and curvature increases, mensici penetrate further into the pore, **d**–**i** Pc $=$ 5000–10,000 Pa, spheres continue to penetrate further but some now reach maximum curvature (red). Not all spheres transition from blue to green before going red and represent a burst before coalescence regardless of phase occupancy

The experiments of Gostick et al. (2009), which we are attempting to simulate here, involved the use of a hydrophobic membrane at the top of the sample, a PTFE gasket contacting the edges and a hydrophilic membrane at the bottom providing the water injection point. On withdrawal, water can only escape from the bottom but not the edges; thus, water that is disconnected from the bottom becomes trapped. Trapping inside single isolated throats is prohibited in the present model by considering the stability of the interface, as explained in Sect. 2.4.5.

#### Cooperative Pore Filling

Cooperative pore filling is a familiar concept in network modelling where the invasion pressure for a given pore is typically scaled down based on the number of connected throats that have access to the invading phase Patzek (2001), Blunt (1997b) and Blunt (1997a). The toroidal model developed in this work allows for a more detailed model which depends on the shape and size of pores, the sizes of throats and the contact angle. As a pre-process to the invasion percolation algorithm, the coalescence capillary pressure is calculated from geometric principles as follows: Filling angles are back-calculated from the toroidal model in all throats for capillary pressures ranging from 0 to 30 kPa at 500 Pa intervals. From the filling angle, the mensici radius of curvature, $r_m$, is also readily determined from the toroidal model Mason and Morrow (1994). Menisci are modelled as spheres with centres outside the pore, and each sphere is assessed for intersection with any of the others. The distance of each meniscus centre relative to the throat centre along the throat normal vector is calculated as:

13 $$\begin{aligned} d_m= R \hbox {sin}(\alpha )-r_m \hbox {cos}(\theta - \alpha -\pi /2) \end{aligned}$$

With incrementing pressure, an analysis is performed for each pore in the network where each throat connecting to the pore potentially facilitates simultaneous invasion from the neighbouring pores. The process is pictured in Fig. 7 where blue spheres are still able to increase pressure and penetrate further, green ones have coalesced and will cooperatively fill the pore and red spheres have reached maximum curvature and will burst into the pore. The first step is a simple check that the distance between any two menisci sphere centres is less than their combined radii. However, the sphere intersection may be outside the pore which does not represent the real menisci that form the spherical caps inside the pore. A second step is required to compute the points of each intersection which form circular planes and to check whether any part of the plane lies within the pore. The lowest pressure in the invading phase for which a valid intersection occurs is recorded in a sparse 2-dimensional square matrix which has dimensions equal to the number of throats in the network. The cooperative pore filling matrix is then used as a reference in the invasion percolation algorithm once throats become accessible to the invading fluid. Cooperative pore filling pressures are generally lesser in magnitude than burst pressures that occur at maximum curvature. However, if a throat is small compared with its immediate neighbours, then maximum curvature may be reached before an intersection occurs. This is depicted in Fig. 7 as spheres turning from blue to red before potentially turning green.

#### Snap-Off

Traditional models of imbibition assume that films can provide unlimited access to the wetting phase throughout the network. In a sense, all the pores are already partially invaded by the wetting phase. In this case, the wetting phase can grow independently from the bulk invasion process and fully invade pores and throats throughout the network. This process has been termed mixed percolation and has been observed and modelled by Ioannidis et al. Ioannidis and Chatzis (1993) in glass micromodels with highly non-wetting fluids, in quasi-2D geometries. As well as changing the percolation pattern, unlimited access for the wetting phase also leads to a phenomenon known as snap-off. The wetting phase grows inside a throat and snaps the non-wetting phase in two leading to disconnection between neighbouring pores and increased levels of trapping.

Ransohoff et al. (1987) consider snap-off in smoothly constricted non-circular capillary tubes and present the following inequality to describe the condition for snap-off.

14 $$\begin{aligned} C_{mc}^* \le \frac{1}{R_C} -\frac{1}{R_{\lambda (0)}} \end{aligned}$$

where $C_{mc}^*$ is a critical curvature of menisci in neighbouring pores, $R_C$ is the smallest radius of the capillary tube at the apex of the constriction corresponding to a throat radius, and $R_{\lambda (0)}$ is the transverse radius of curvature of the constriction corresponding to a fibre radius in the present model. As GDLs typically have a fibre radius of 5 $\upmu$m and throat radii between 5 and 50 $\mu$m, the condition necessitates that curvature (and therefore capillary pressure) in the pore become zero for the smallest throats and highly negative for larger throats before snap-off may happen. Equation 14 was derived for perfectly wetting fluids which may easily reside in the corner features of throats. In the present study, we apply the snap-off criterion but only when the throat geometry permits the formation of multiple arc menisci which can grow and coalesce. Fortunately, the Voronoi diagram provides the information required as every throat is planar and defined by a set of vertices which form the fibres. It is therefore possible to calculate the throat corner angles, $\beta$, and apply the following rule for the formation of arc menisci Hoiland et al. (2007):

15 $$\begin{aligned} \theta \le (\pi - \beta )/2 \end{aligned}$$

#### Interface Stability

A scenario can occur where defending phase becomes isolated inside a single throat and must be given special consideration to become invaded. As pressure increases in the invading phase, filling angles increase and the meniscus on either side penetrates further towards the opposing side of the throat. At some filling angle, the two mensici will touch and coalesce and the defending phase will break apart. This will happen at a lower capillary pressure than for standard “burst” invasion which requires the meniscus to advance past the throat apex. Although water is considered incompressible, the irregular shape of the throats in the network allows for redistribution of fluid and menisci touching is considered highly probably for throats that have radii comparable to the fibre radius and larger.

Rather than explicitly calculating the threshold pressure where breakup occurs for every throat in the network, such throats are simply invaded instantaneously when isolation first occurs. In effect, the condition means that the trapping rules only apply to pores and throats connecting trapped pores. Invading isolated throats was found to have a negligible effect on the characteristic capillary pressure curves, as the throats do not contribute much to the overall saturation. However, the condition has a marked effect on the relative diffusivity curves, reducing the tortuosity of the invading phase connected pathways significantly. Therefore, network simulations which do not consider the instability of such interfaces may significantly under-predict relative transport through such fibrous media.

**Fig. 8:**
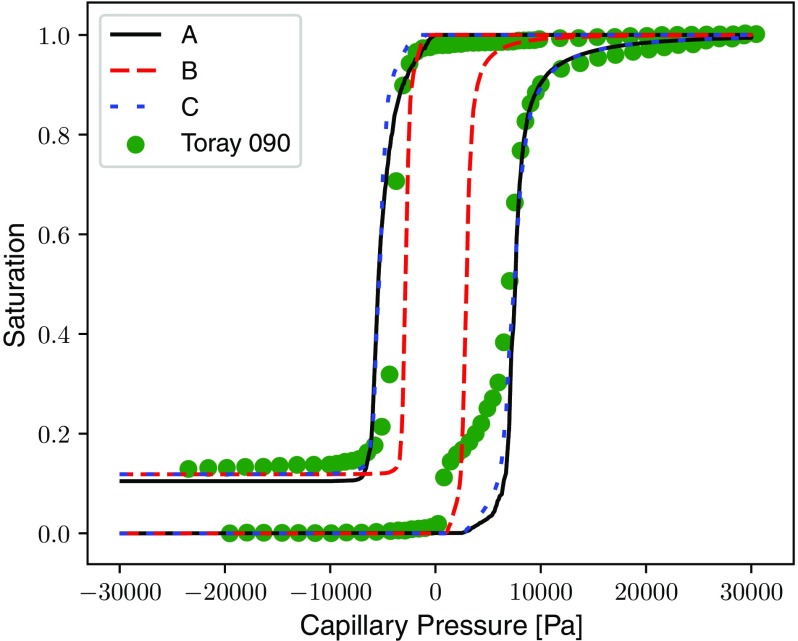
Saturation versus Capillary Pressure generated by the PNMs using the invasion percolation with trapping model. Compared with experimental data collected by Gostick et al. for uncompressed Toray 090 GDL with 20% PTFE treatment Gostick et al. (2009). Case details are provided in Table 1

## Results and Discussion

### Capillary Pressure Hysteresis

In this section, numerical results generated for each case using the two capillary pressure models are compared with experimental data collected by Gostick et al. (2009) for the uncompressed Toray 090 sample treated with 20% PTFE by weight. As described in Sect. 2.3.2, the network was tuned to achieve the best fit with the water injection data, porosity and permeability. Figure 8 shows the network saturation as a function of capillary pressure for each case compared with the experimental data. Good agreement is found between simulation and experiment when using the toroidal model in Case A. However, Case B with moderate hysteresis using the Washburn model under-predicts the capillary pressure for a given saturation. Case C with contact angle hysteresis of 100$^\circ$ must be employed with the Washburn model to get the negative water withdrawal pressures and unreasonably high contact angles must be used to match the magnitude of the water injection pressures. The “shoulder” in the experimental data at low positive capillary pressure at the beginning of the water injection is an artefact of the imperfect contact between the GDL sample and the hydrophilic membrane and disappears when sample compression is increased.

**Table 3:** Percentage occurrence of invasion actions for the injection and withdrawal of water for Case A

Invasion action	Injection (%)	Withdrawal (%)
Burst	79	52
Touch	17	20
Cooperative Fill	4	26
Snap-off	0	2

Differences arise because the air phase penetrates further into the pore before reaching maximum curvature, leading to greater interaction with pore walls and other menisci

Table 3 shows the percentage of invasion actions that took place during each stage of the simulation for Case A. Cooperative pore filling occurs much more frequently during water withdrawal as air reaches a greater filling angle and penetrates further into the pore before reaching maximum curvature. This reduces the magnitude of the invasion pressure in the air phase and also leads to a slightly more frontal advance.

It was explained in Sect. 2.1 that a positive pressure is always required in the invading phase to penetrate through a toroidal constriction, irrespective of the contact angle. This offers an explanation that hysteresis in the capillary pressure data does not result purely from contact angle hysteresis and also explains why spontaneous uptake of neither water nor air is seen at the beginning of experiments. There has been no explanation given for contact angle hysteresis inside the porous structure of a GDL, especially one that would give rise to such a drastic change in wettability, as modelled using Eq. 1 for Case C.

Contact angle hysteresis is generally understood as a surface phenomenon observed in droplets under stress where the advancing contact angle is greater than the receding and can be attributed to pinning of the trailing contact line Gao and McCarthy (2006) by rough surface features. To the authors’ knowledge, there is no visualisation of contact angle hysteresis occurring for droplets inside the GDL. Gurau et al. have measured the static internal contact angle with a combination of the Washburn and Owens-Wendt methods Gurau et al. (2007) but can only report average values. Experimental observations of contact angle hysteresis have been performed using roughened capillary tubes by Morrow Morrow (1975) and Raeesi et al. (2013). In these experiments, capillary rise is used to measure an “apparent” contact angle which is inferred using the Washburn model. With spiral roughening in a lateral direction to the meniscus movement, an extreme hysteresis of 140$^\circ$ was observed for otherwise neutrally wetting fluid pairs Raeesi et al. (2013). This roughening would have presented the interface with a series of undulating grooves similar in shape to a fibrous surface and so may also be explained by a converging–diverging type effect.

Two scales of “roughness” can be considered present in the GDL, a microscale roughness caused by the curvature of the fibres and a sub-microscale roughness at the surface of the fibres, binder and coating. Both may modify the “apparent” contact angle when employing the Washburn model. However, this representation is convoluted and not necessarily helpful for material design. The present study focuses on the curvature of the fibres and reproduces the capillary characteristics entirely without employing contact angle hysteresis attributed to nanoscale roughness by considering a more realistic alternative to the Washburn model in Case A. The surfaces of the fibres are not perfectly smooth, but the overriding feature of the local pore-scale geometry is the curvature of the fibres. Experimental results are also being collected for Freudenberg GDLs which have very smooth fibres and are displaying the same characteristic hysteresis which will be published at a future date.

The idea of contact angle hysteresis is potentially misleading for engineers wishing to change the properties of fibrous materials. Applying hydrophobic treatment has the effect of increasing the intrinsic contact angle slightly, but water withdrawal is still unfavourable at positive capillary pressure. The present study suggests that contact angle is not the dominant factor determining the capillary pressure characteristic, local pore-scale geometry is. Furthermore, modifying the material structure in ways to negate the effect of the converging–diverging fibres could have benefits for water management. For example, alternatives to fibres or perhaps differently shaped fibres which do not present a capillary barrier could result in greatly improved water management in GDLs.

It is recommended based on these results that the simple Washburn model should not be used to interpret capillary behaviour in fibrous media as either contact angles will be over-predicted or pore sizes will be under-predicted. Moreover, the toroidal model is physically satisfying as the constrictions between fibres found in the GDL are much more like the inner surface of a torus, rather than a straight capillary tube, and the hysteresis in phase pressure is a natural consequence of the local solid geometry.

### Implication for Fuel Cells

In an operating fuel cell, water clusters may be in an injection or a withdrawal configuration or a combination of both, depending on operating conditions and power requirements. Liquid withdrawal characteristics are important when drying occurs and the liquid distribution may be significantly different from an injection configuration which may occur during start up or periods of high power production. Understanding these difference is important for predicting fuel cell performance as diffusional mass transport can become severely limited by water blockages and without sufficient diffusion the catalyst layers cease to be supplied with reactive species. In this section, results are presented for saturation distributions determined using the toroidal capillary pressure model without trapping as isolated clusters are assumed to evaporate over time. To simulate the conditions under which relative diffusivity experiments have been conducted, both top and bottom faces of the domain are used to select boundary pores for invasion. For comparison, a single face is also used to apply invasion inlets as this may more closely resemble the operating fuel cell under certain conditions. The bottom face is used for water injection and top for withdrawal.

#### Relative Diffusivity

The relative diffusivity is an important parameter for multiphase characterisation of porous media and is indicative of how well phases are separated and consequently how tortuous the gas pathways are. A linear relation between effective diffusivity and saturation indicates well separated phases with the connected pathways experiencing little tortuosity, thus acting like straight gas channels though the water. A power law dependence is more common where higher exponents indicate more tortuous flow paths.

**Fig. 9:**
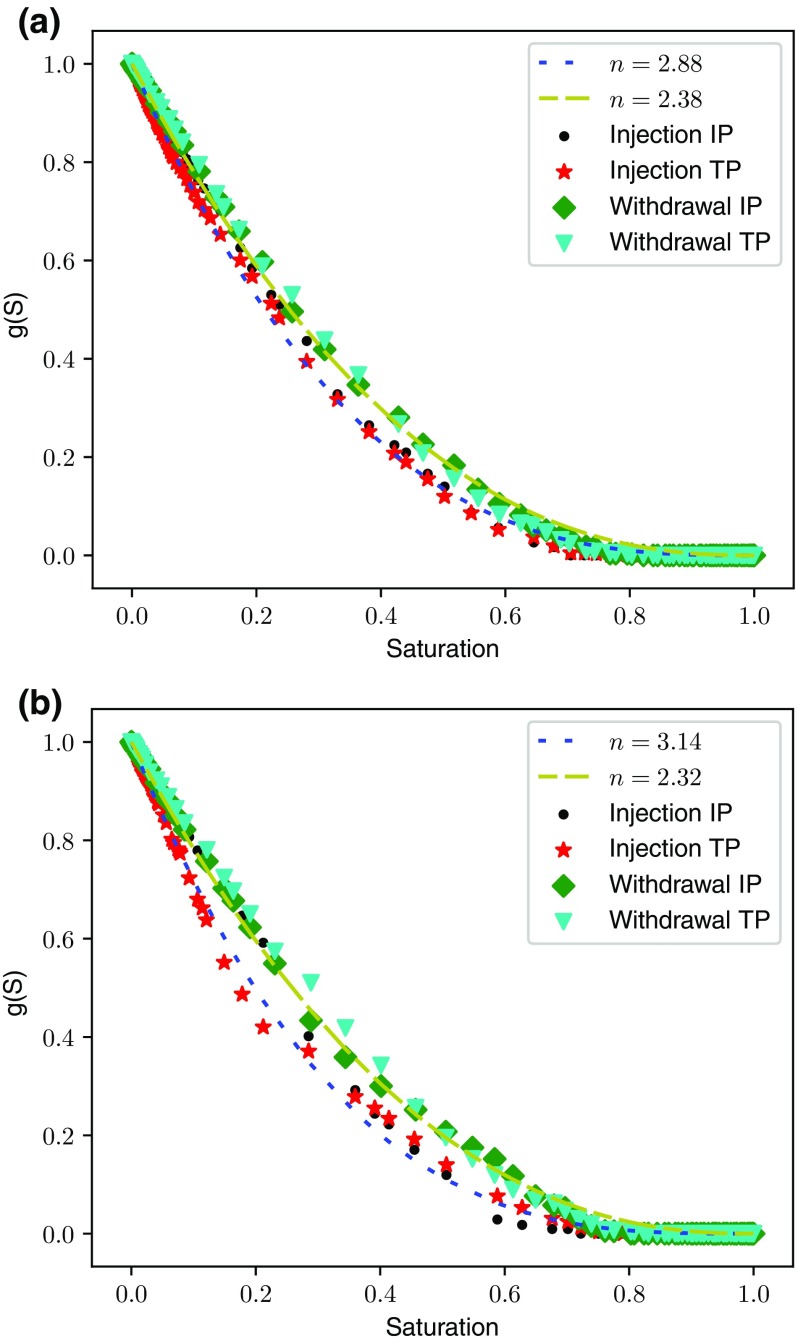
Relative diffusivity of air calculated from the phase distributions determined by injection and withdrawal using Case A parameters without trapping: **a** inlets applied to both boundaries, **b** inlets applied to a single boundary

Results were obtained by calculating the diffusivity as described in Sect. 2.3.1 in the gas phase at varying levels of water saturation set by the percolation algorithm. The absolute effective diffusivity of the dry network was calculated, and presented in Sect. 2.3.2, and this value is used to normalise the results for partial water occupancy at each saturation enabling the relative air diffusivity to be calculated using Eq. 9. The results are presented in Fig. 9 and tend to follow a power law with values of *n* presented in the figure legends:

16 $$\begin{aligned} g(S) = (1-S)^n \end{aligned}$$

The power law is a good fit up to saturations of 0.8 but less so thereafter where no percolating clusters are present and diffusion drops to zero. A clear trend is that the power exponent tends to be higher for water injection than for withdrawal. Comparing Fig. 9a with b, invasion from a single face leads to a slightly higher power than invasion from both faces for injection and a slightly lower power for withdrawal. However, there is little difference overall between the simulations with differing boundary conditions, which at first glance is surprising but will be explained by the saturation distribution. The range of values for the exponent agrees fairly well with Lattice–Boltzmann simulations García-Salaberri et al. (2015b) and García-Salaberri et al. (2015a) reporting exponents between 2 and 3, depending on the uniformity of the saturation distribution. In addition, our recent experiments for the in-plane diffusivity also report power law behaviour between 2 and 2.5 Tranter et al. (2017). Interestingly, although the dry networks exhibit significant anisotropy in absolute transport behaviour, the relative transport follows an almost identical pattern for both IP and TP directions. The exception being injection from a single boundary which favours IP transport at saturations below about 0.3 as most of the saturation resides near the bottom face, creating a bottle-neck for TP transport.

**Fig. 10:**
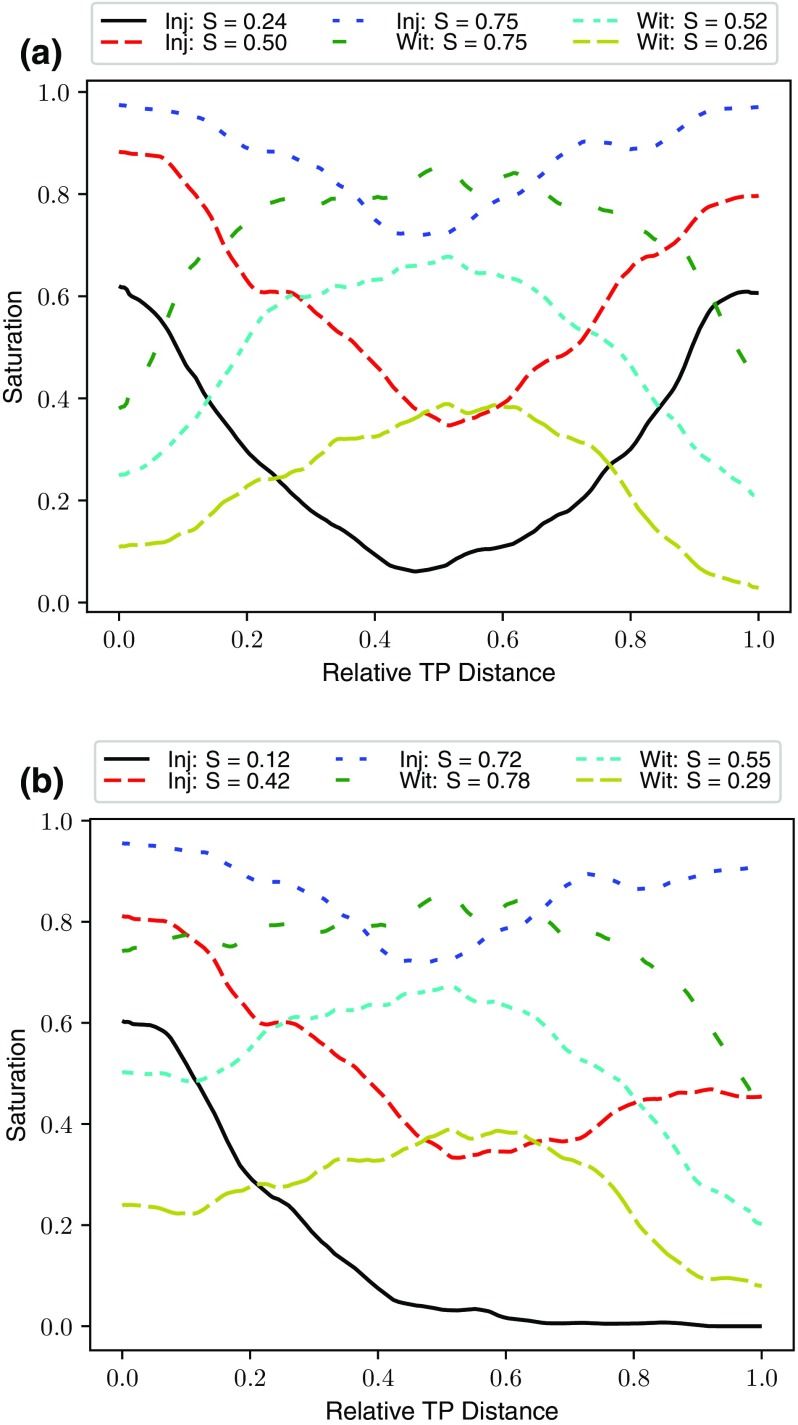
Saturation profile as a function of through-plane domain position determined by injection and withdrawal using Case A parameters without trapping: **a** inlets applied to both boundaries, **b** inlets applied to a single boundary. Although profiles are different between **a**, **b** the peak saturation is similar

Figure 9 shows that for saturations below the percolation threshold, diffusion is better through the air phase when air is invading. At intermediate saturation, the largest difference is about a 50% increase in air diffusivity under air invasion. This is a key finding since it suggests that when a fuel cell is drying it will operate significantly better, compared with one that is undergoing water injection as the primary filling mechanism, despite having the same overall saturation. This result implies that fuel cell performance is saturation-history dependent. The difference in characteristic diffusivity between water injection and withdrawal arises from the diffusing air phase taking on different roles during each scenario, defender or invader. Invasion proceeds in both scenarios on a largest throat first basis. The larger throats accessible to the invading cluster will generally be connected to larger pores and so these also fill with invading fluid before smaller pores and throats. Therefore, the air phase conductivity is generally better when air is invading as it occupies a greater proportion of larger pores and throats. For air invasion, low liquid saturation occurs at the end of the invasion process, and the liquid is residing in a number of smaller pores and throats, randomly distributed in the network, not affecting the overall air connectivity or conductivity. Conversely for water invasion, the largest pores and throats in the air-filled network are invaded and “knocked-out” of the air-filled network first, consequently diffusion suffers more.

#### Effect of Saturation Distribution

The network generation technique used in the present study is based on the creation of an image from a Voronoi diagram as detailed in Sect. 2.2. Once the percolation algorithms have run, a list of invaded pores for any given saturation is available and their identifying indices can be used to back-populate the original image of the fibres to simulate a realistic multiphase configuration as shown in Fig. 6. In doing so, detailed saturation distributions may also be generated from the images and are provided in Fig. 10a, b for both sets of boundary conditions. An interesting observation can be made, which is that although the saturation profiles are quite different, the peak saturation is similar for both boundary conditions. This fact explains why no great difference in relative diffusivity is recorded between the simulations and reinforces the observation made by García-Salaberri et al. that peak saturation is more important than average saturation for determining overall transport behaviour García-Salaberri et al. (2015b) and García-Salaberri et al. (2015a).

## Conclusion

A scheme for generating pore network models of high porosity fibrous media was presented. This model was successfully used to describe the capillary pressure characteristics of a typical fuel cell gas diffusion layer. Using Purcell’s toroidal model for capillary entry pressure with modifications to account for phase interactions away from the throat apex enabled the matching of both water injection and withdrawal data in fibrous media for the first time. The toroidal model does not require employing dubious assumptions about contact angle hysteresis as a fitting parameter, as the Washburn model does. The toroidal model is therefore more reliable when inferring pore-size distribution from capillary pressure characteristic data and for subsequently performing multiphase simulations.

The toroidal capillary pressure model was combined with considerations of the surrounding pore geometry and produces bi-directionality of the invasion algorithm. A novel cooperative pore filling method was also introduced, again using the toroidal geometry as the fundamental construct, and fluid topology was found to influence water withdrawal significantly. Withdrawal of water is modelled as a forceful injection of air, following the same percolation rules as injection of water, as neither fluid is truly wetting in the traditional sense. However, contact angle plays a part in determining the pore penetration of menisci which affects the frequency of different invasion mechanisms. Pore geometry has been shown to play a more important role than contact angle in determining average injection and withdrawal pressures for neutrally wettable fibrous media. Furthermore, results suggest that pore structure should be the focus of future efforts to control the multiphase characteristics of fibrous media, with network modelling playing a key role.

Relative diffusivity of the networks was shown to match well with the literature under different percolation scenarios, and it is demonstrated that care should be taken when using constitutive relations to consider the effects of phase configuration as peak saturation is a more important parameter. One constitutive relation may not be sufficient to cover all operating scenarios when continuum modelling fuel cells, and it is recommended to use pore network modelling to establish the phase configuration using the toroidal model, which can now be applied to both injection and withdrawal of water.

## Data Statement

All of the data used in this study are included in this paper.
